# The development and validation of a machine learning algorithm for identifying lateral lymph nodes skip metastasis in papillary thyroid cancer

**DOI:** 10.3389/fonc.2026.1715450

**Published:** 2026-03-26

**Authors:** Gong Cheng, Xueming Zhang, Lixin Cheng, Yi Ruan, Yu Qiu

**Affiliations:** 1 Department of Hepatobiliary Pancreatic Surgery, Ningbo Medical Center Lihuili Hospital, Ningbo, Zhejiang, China; 2 Head and Neck Surgery, Ningbo Medical Center Lihuili Hospital, Ningbo, Zhejiang, China

**Keywords:** lateral lymph node metastasis, machine learning, papillary thyroid carcinoma, random forest method, skip metastasis

## Abstract

**Background:**

Skip metastasis from papillary thyroid cancer (PTC) is often unpredictable and characterized by lateral lymph node metastasis without central lymph node metastasis. Our objective was to provide a predictive model for skip metastases to cervical lymph nodes based on clinical and demographic data using machine learning.

**Materials and methods:**

From January 2016 to December 2021, patients who underwent thyroidectomy, central lymph node dissection, and lateral lymph node dissection at the Department of Thyroid Surgery at our hospital, had their clinical and pathological data analyzed retrospectively. Following the identification of five critical characteristics, six machine learning models were developed. Accuracy, sensitivity, specificity, positive predictive value, negative predictive value, F1 score, kappa statistics, and area under the curve were measured in the performance evaluation process, and decision curve analysis was used to determine the clinical advantage. Next, universality was assessed through internal validation. R and Python software was used for all statistical analyses and model construction.

**Results:**

The incidence of skip lymph node metastases was 13.02% (47/361). Pertinent elements encompassed the number of nodes removed as a result of central lymph node dissection, the existence or non-existence of Hashimoto thyroiditis, the largest tumour size, its bilateral nature, and its multifocal nature. By outperforming alternative models, the random forest model demonstrated excellent performance on the internal validation cohort.

**Conclusion:**

This study focused on identifying the risk factors associated with skip metastasis, with the aim of developing an efficient predictive model for this condition using readily available clinical variables. This model can precisely identify skip metastases in PTC using an uncomplicated approach, offering promise for routine clinical use.

## 1 Introduction

Thyroid carcinoma is one of the most common endocrine malignancies (1, 2). Owing to advancements in ultrasound-guided puncture and high resolution ultrasonography (3), together with the increasing awareness of thyroid carcinoma, its prevalence has skyrocketed globally in recent years (4). Approximately 90% of thyroid cancers are papillary thyroid carcinomas (PTCs), which are regarded as low-risk malignancies with favorable prognoses (5). Early lymph node metastasis is common, and increases the possibility of recurrence and reoperation (6, 7). First, lymph node metastases occur in the regional nodal basins of the neck, with approximately 20–30% occurring in the lateral compartment (levels II–V) and 50–60% in the central compartment (levels VI–VII) (8). Compared to patients with lymph node metastasis in the central area (central lymph node metastasis, CLNM), those with lateral lymph node metastasis (LLNM) have poorer outcomes, with a higher rate of recurrence and distant metastases (9, 10). While compartment-oriented lymph node dissection is necessary, there is disagreement regarding which individuals with PTC truly need neck dissection (11–13).

Prophylactic lateral neck dissection is not recommended for patients with a clinically negative (cN0) lateral neck unless preoperative fine-needle aspiration cytology confirms a suspicion of LLNM (14, 15). The two main techniques for evaluating lymph nodes preoperatively are computed tomography and ultrasonography; however, their detection accuracy is limited (16, 17). The sonographic features of most metastatic lymph nodes are atypical, particularly in early stages (18, 19).

Predicting lymph node metastasis before surgery can help determine the necessary scope of surgery, select the optimal surgical approach for each patient, support removal of all metastatic lymph nodes, lower the likelihood of repeat surgery, and increase patient survival and prognosis. It is possible to limit the occurrence of surgical problems such as hypoparathyroidism, recurrent laryngeal nerve injury, and vocal nerve injury by minimizing unnecessary prophylactic lymph node dissection (20, 21).

LLNM typically follows CLNM, owing to the morphology of the lymphatic drainage system (22, 23). When metastasis to the lateral compartment occurs without the involvement of the core lymph node compartment, it is referred to as skip metastasis. Skip metastases can occur in 1.6–25% of patients with LLNM (24–26). Previous studies have established several predictive models for risk of skip metastasis that distinguish patients with skip metastasis from those with CLNM and LLNM (27, 28); however, their practical clinical utility remains restricted. This study aimed to provide a more precise depiction of the clinical features of skip metastases, drawing on prior studies, and to create a prediction model via machine learning that is more applicable in clinical settings. Such a model can lessen the chances of reoperation and postoperative recurrence, and spare low-risk patients from the psychological and financial strain resulting from overtreatment.

## 2 Materials and methods

### 2.1 Patient cohort and surgical procedure

This study has been approved by our hospital’s ethics committee. As this is a retrospective study conducted in a single center, the ethics approval number is: KY2025SL423-01. This study utilized the clinical data of PTC patients who visited our hospital from January 2016 to December 2021.

The inclusion criteria were as follows: (1) age ≥18 years; (2) available preoperative high-resolution thyroid ultrasound scan, with comprehensive image records to assess the cervical lymph node status; (3) history of extensive lymph node dissection and at least a single unilateral thyroid lobectomy; and (4) pathologically diagnosed PTC, with a pathological report of cervical lymph nodes and more than 0 excised cervical lymph nodes. The exclusion criteria were as follows: (1) previous or concurrent malignant tumors; (2) history of prior treatment; (3) secondary thyroid surgery or without regional lymph node dissection; or (4) incomplete clinical data. Patients who underwent CLND alone or LLND alone were excluded from this study, as the diagnosis of skip metastasis requires pathological evaluation of both central and lateral compartments. In total, 361 individuals were enrolled in this study (**Table 1**).

**Table 1:** Demographics and clinical characteristics of the cohort.

Variable	Cohort
	Overall, N =361
**Age, M (Q_1_, Q_2_)**	43.00 (33.00, 53.00)
Sex	
Male	126 (34.90)
Female	235 (65.10)
**MTD, M (Q_1_, Q_2_)**	1.20 (0.80, 2.00)
Multifocality	
Absent	219 (60.66)
Present	142 (39.34)
Bilaterality	
Absent	233 (64.54)
Present	128 (35.46)
Hashimoto’s thyroiditis	
Absent	305 (84.49)
Present	56 (15.51)
Nodular goiter	
Absent	246 (68.14)
Present	115 (31.86)
Extension	
intrathyroidal	252 (69.81)
extrathyroidal	109 (30.19)
TG, M (Q_1_, Q_2_)	2.13 (1.04, 19.00)
LNM	
Without LNM	20 (5.54)
CLNM only	20 (5.54)
LLNM only	47 (13.02)
CLNM and LLNM	274 (75.90)
CLNM number	3.00 (1.00, 5.00)
CLND number	6.00 (3.00, 9.00)
LLNM number	3.00 (2.00, 6.00)
LLND number	23.00 (16.00, 31.00)

M: Median, Q_1_: 1st Quartile, Q_2_: 3st Quartile.

MTD, maximum tumor dimension; TG, thyroglobulin; LNM, lymph node metastasis; CLNM, central lymph node metastasis; LLNM, lateral lymph node metastasis; CLND, central lymph node dissection; LLND, lateral lymph node dissection.

All data were manually extracted and cross-verified by two independent investigators to ensure completeness and accuracy. To minimize selection bias, no additional case selection was performed beyond the defined criteria, and all eligible patients during the study period were included consecutively.

Skip metastasis was operationally defined as histopathologically confirmed lateral lymph node metastasis (LLNM) in the absence of any central lymph node metastasis (CLNM), based on postoperative pathological examination of both central and lateral compartment dissection specimens. Patients with positive central lymph nodes, regardless of lateral compartment status, were not classified as skip metastasis.

The lymph nodes were identified solely by the surgeon. Concurrent thyroidectomy and cervical lymph node dissection, lateral lymph node dissection (LLND) or central lymph node dissection (CLND), were performed. Initial surgery always included dissection of the central lymph nodes. Preoperative neck mapping was performed using cervical ultrasonography and enhanced computed tomography. When lateral compartment lymph node metastases were suspected on imaging or verified by fine-needle aspiration biopsy, patients underwent dissection of area II, III, IV, and Vb.

### 2.2 Statistical analysis

Descriptive statistics were used for baseline data and features of patients with PTC. Categorical variables are presented as numbers and percentages. Using a randomization method, 70% of the study population was assigned to a training cohort for model development and 30% into the validation cohort.

Choosing the right features plays a crucial role in developing a machine learning model as it pinpoints the most pertinent feature subset, greatly enhancing classification precision and minimizing model overfitting. Features deemed significant or provisional were selected using the Boruta method (29, 30). Boruta’s approach employs a random forest (RF) method to select features for identifying all pertinent characteristics linked to the response variable. The process identifies significant features through RF training of the dataset, contrasting the initial significance of these features against the “shadow features” significance derived by randomly rearranging their sequence.

Prior to applying the Boruta algorithm, we constructed an initial feature pool comprising 11 candidate variables. These variables were selected based on: (1) systematic review of the literature on risk factors for PTC lymph node metastasis and skip metastasis[24–28]; (2) clinical availability in routine preoperative and postoperative settings; and (3) input from experienced thyroid surgeons at our institution.

We selected six common classification models to build machine learning models for predicting lymph node skip metastasis: logistic regression (LR), extreme gradient boosting, RF, adaptive boosting, support vector machine, and K-nearest neighbor (KNN) models. Every set of parameters underwent ten rounds of cross-validation, choosing the hyperparameter set that excelled in performance and setting the minimal logarithmic loss as the reference for this study. Subsequently, the ultimate machine-learning model was established by thoroughly retraining the complete training dataset.

Following the construction of our machine learning model, multiple evaluation dimensions were used to assess the model performance, including accuracy, sensitivity, specificity, positive predictive value, negative predictive value (NPV), F1 score, Kappa statistics, and area under the curve (AUC). The closer the accuracy, AUC, sensitivity, and specificity are to 1, the better the model’s performance. A decision curve analysis (DCA) was performed to assess the therapeutic advantages of the model. The net benefit of different models in predicting skip metastasis of lateral cervical lymph nodes under different threshold settings was used to further guide the optimization of clinical decision-making.

We evaluated the same criteria that were used for model comparison and then evaluated the top-performing model on an internal validation queue.

To develop and evaluate our prediction models, the dataset was randomly divided into a training cohort (70%) and a validation cohort (30%). During model training on the training cohort, 10-fold cross-validation was employed for hyperparameter tuning to prevent overfitting. The final model performance was then assessed on the independent validation cohort. The training set and the validation (or internal validation) set are two of the ten subsets of approximately similar size that make up the available training sets. One of them was used to gauge the model’s accuracy, while the other nine were utilized to fit the model.

The statistical analysis above was conducted using R software (http://www.r-project.org/) and Python scripting language (Version 3.6.5, Python Software Foundation, Wilmington, DE, USA, https://www.python.org).

## 3 Results

### 3.1 Demographics and characteristics of patients

The characteristics of the patients are presented in **Table 1**. In total, 321 (88.9%) patients had LLNM and 47 (14.0%) had skip metastases. In patients with skip metastases, CLNM, and LLNM, the average number of harvested central lymph nodes was 15.5, 6.8, and 24.2, respectively. The average number of positive central lymph nodes in patients with CLNM and LLNM was 3.5 and 4.2, respectively (**Table 1**).

Based on the extent of cervical metastasis, the patients were categorized into three groups (**Table 2**): SKIP group (n = 47 patients with skip metastases), CLNM group (n = 20 patients with CLNM only), and LLNM group (n = 274 patients with CLNM and LLNM). Regarding tumor size, patients in the SKIP group had significantly smaller tumors (mean 0.9 cm, range 0.3-2.1 cm) compared to both the CLNM group (mean 1.6 cm, range 0.5-3.2 cm; P = 0.009) and the LLNM group (mean 1.4 cm, range 0.4-3.5 cm; P = 0.02), indicating that skip metastasis is associated with smaller primary tumors. For tumor bilaterality, the SKIP group showed a significantly higher proportion of bilateral tumors (36.2%, 17/47) compared to the CLNM group (10.0%, 2/20; P = 0.009) and the LLNM group (19.3%, 53/274; P = 0.022), suggesting that bilateral disease increases the likelihood of skip metastasis. Regarding multifocality, the SKIP group demonstrated a significantly higher rate of multifocal tumors (44.7%, 21/47) than the CLNM group (20.0%, 4/20; P = 0.03). Although the SKIP group also showed a higher multifocality rate compared to the LLNM group (32.8%, 90/274), this difference did not reach statistical significance (P = 0.11).

**Table 2:** The clinicopathological characteristics of PTC patients with LNM.

Variable	CLNM group	P value (Skip vs. CLNM)	Skip group	P value (Skip vs. LLNM)	LLNM Group
	N =294 (%)		N =47 (%)		N =321 (%)
Age, M (Q_1_, Q_2_)	41.50 (32.00, 52.00)	0.367	44.00 (36.50, 52.50)	0.355	41.00 (33.00, 52.00)
Sex		0.552		0.545	
Male	107 (36.39)		15 (31.91)		117 (36.45)
Female	187 (63.61)		32 (68.09)		204 (63.55)
MTD, M (Q_1_, Q_2_)	1.40 (0.90, 2.00)	**0.009**	1.00 (0.65, 1.80)	**0.020**	1.30 (0.80, 2.00)
Multifocality		**0.030**		0.054	
Absent	170 (57.82)		35 (74.47)		192 (59.81)
Present	124 (42.18)		12 (25.53)		129 (40.19)
Bilaterality		**0.009**		**0.022**	
Absent	180 (61.22)		38 (80.85)		205 (63.86)
Present	114 (38.78)		9 (19.15)		116 (36.14)
Hashimoto’s thyroiditis		0.271		0.418	
Absent	44 (14.97)		10 (21.28)		53 (16.51)
Present	250 (85.03)		37 (78.72)		268 (83.49)
Nodular goiter		0.115		0.169	
Absent	91 (30.95)		10 (21.28)		104 (32.40)
Present	203 (69.05)		37 (78.72)		217 (67.60)
Extension		0.175		0.229	
intrathyroidal	196 (66.67)		36 (76.60)		218 (67.91)
extrathyroidal	98 (33.33)		11 (23.40)		103 (32.09)
TG, M (Q_1_, Q_2_)	2.22 (1.00, 19.08)	0.296	1.73 (1.00, 15.05)	0.367	2.08 (0.99, 19.00)

M: Median, Q_1_: 1st Quartile, Q_2_: 3st Quartile.

*MTD*, maximum tumor dimension; *TG*, thyroglobulin.

Bold: P < 0.05.

### 3.2 Feature variable selection using the Boruta method

**Figure 1:**
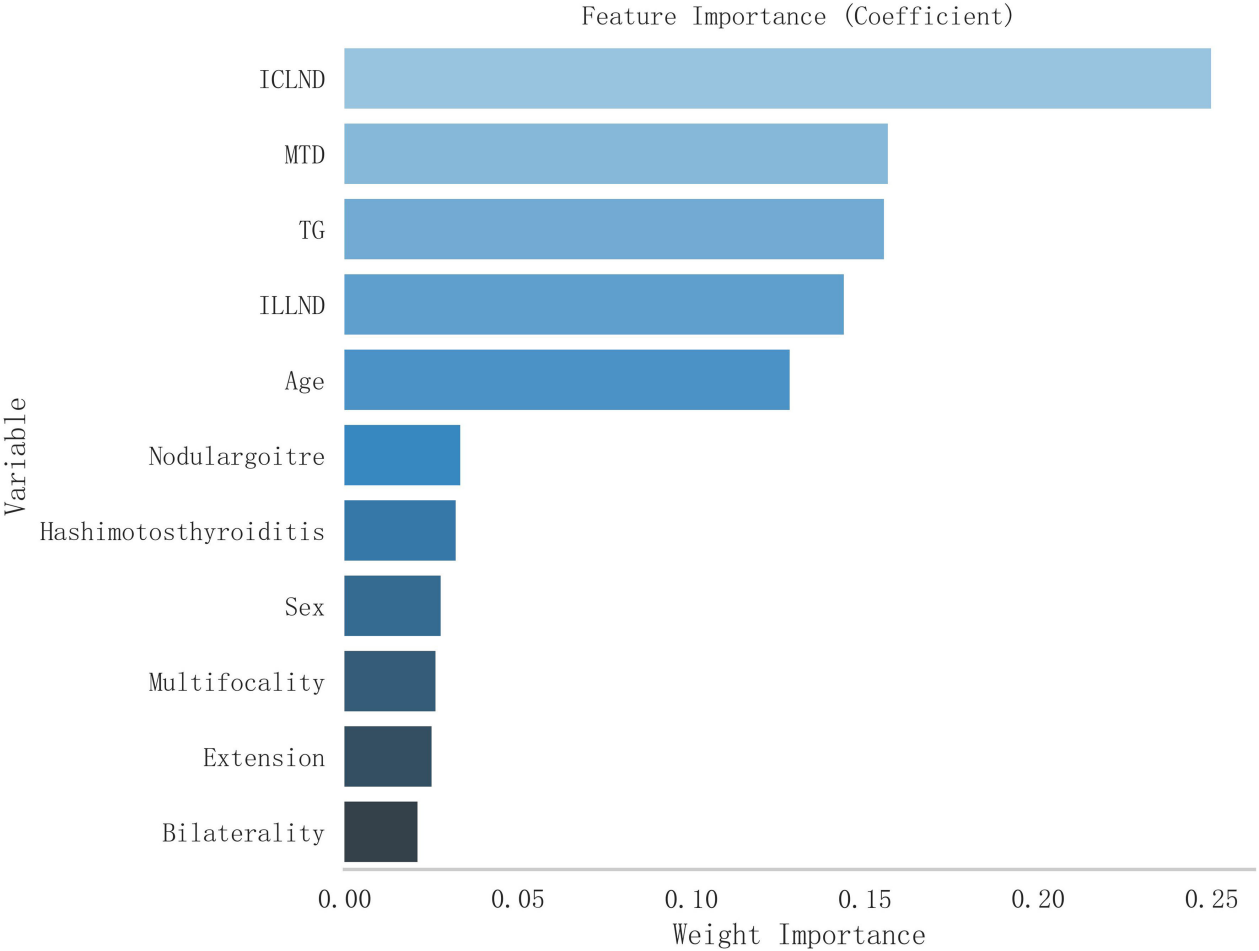
The significance of the eleven factors incorporated into the model used for forecasting skip metastasis in lateral lymph nodes.

Typically, inaccuracies in a model diminish as the selection of variables increases. However, augmenting the number of variables adversely affects clinical applicability. To identify the key characteristics, the RF method was used to select variables through diverse subsets of features. Consequently, attributes based on treetops play a greater role in forecasting the absence of lymph node metastasis in the lateral cervical area of patients with PTC. The significance of each feature is depicted in **Figure 1**.

The Boruta method was employed to identify the characteristics that were considered significant or provisional. Different-colored box plots represent different results of the Boruta feature selection: green indicates that the variable was accepted as a feature, yellow indicates that the variable was leaning towards acceptance, and red indicates that the variable was rejected. The blue box plots represent shadow variables.

**Figure 2:**
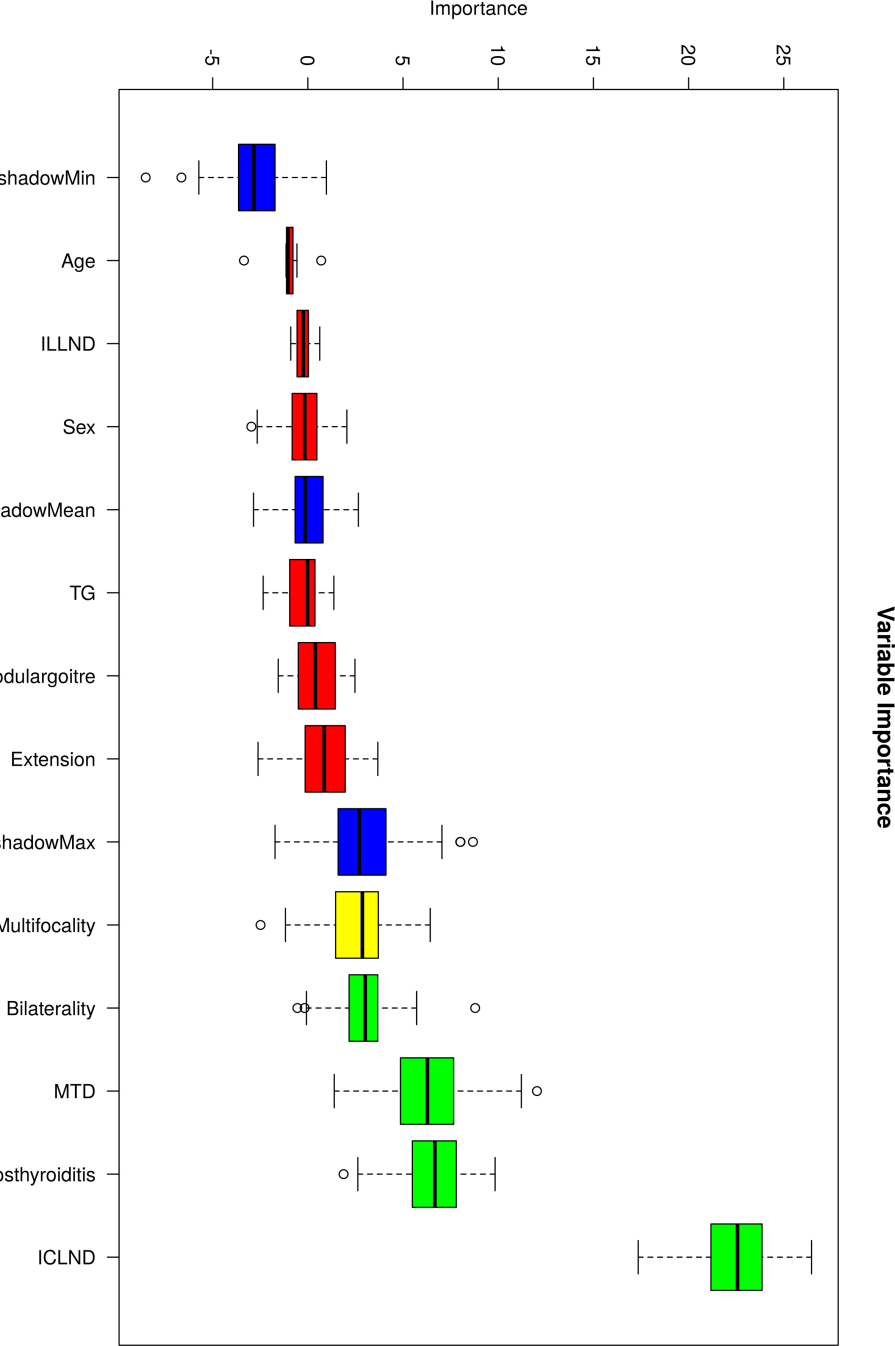
Feature variable selection using the Boruta method.

Based on the above results, we used a combination of five selected features to build a machine learning model: number of removed central neck lymph nodes, Hashimoto’s thyroiditis, presence of multiple tumour sites, presence of bilateral thyroid cancer, and tumour size (**Figure 2**).

Among the five selected features, the number of removed central lymph nodes and Hashimoto’s thyroiditis showed the strongest predictive influence. A lower number of resected central nodes may indicate incomplete sampling or anatomical variations with direct lateral drainage. Hashimoto’s thyroiditis may alter lymphatic drainage patterns through chronic inflammation, disrupting the typical stepwise metastasis from central to lateral compartments. Tumor size, multifocality, and bilaterality likely reflect tumor biology that enables access to alternative lymphatic pathways bypassing the central compartment.

### 3.3 Performance of machine learning algorithms

**Figure 3:**
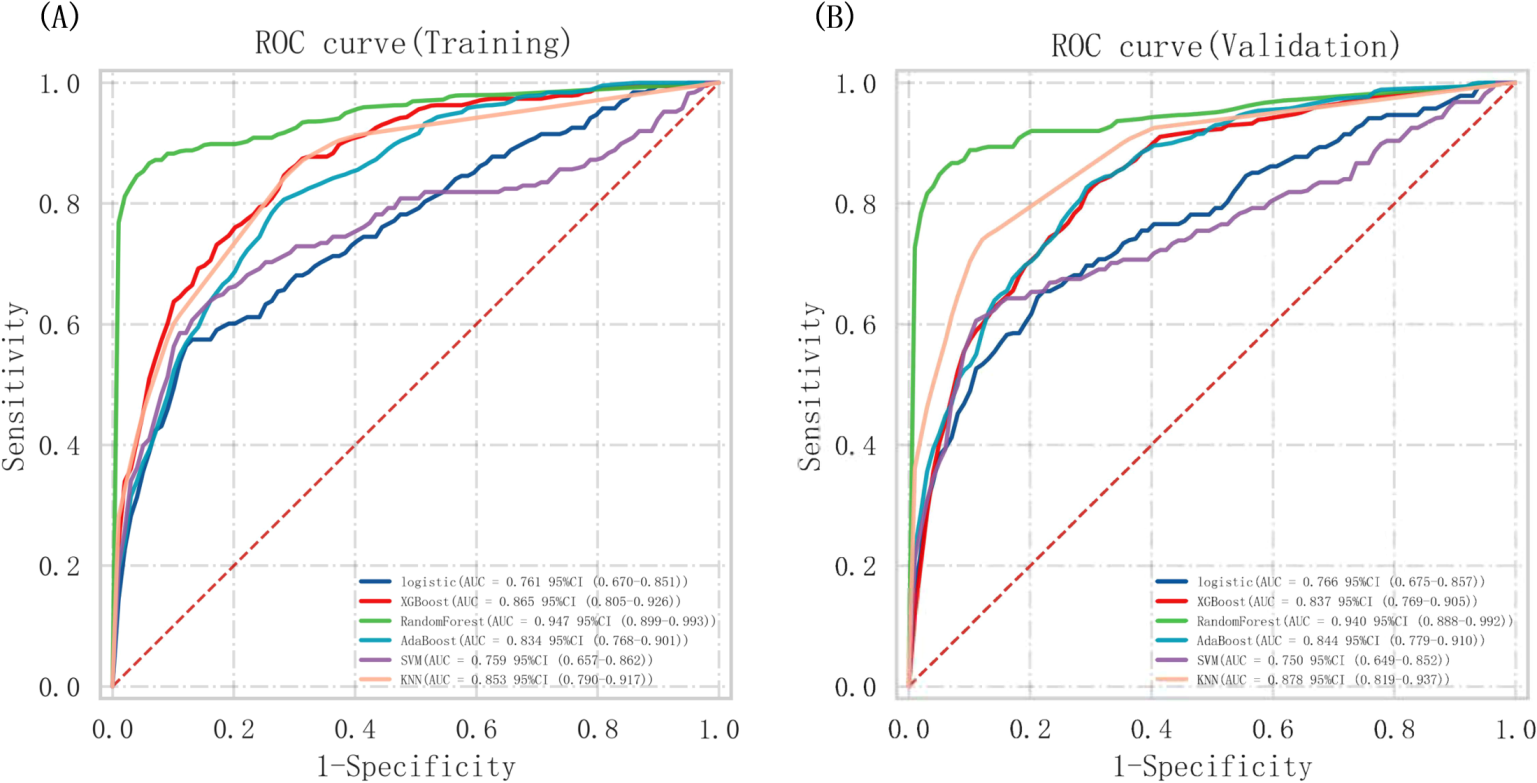
ROC curves of all models **(A)** Training cohort **(B)** Validation cohort.

**Figure 4:**
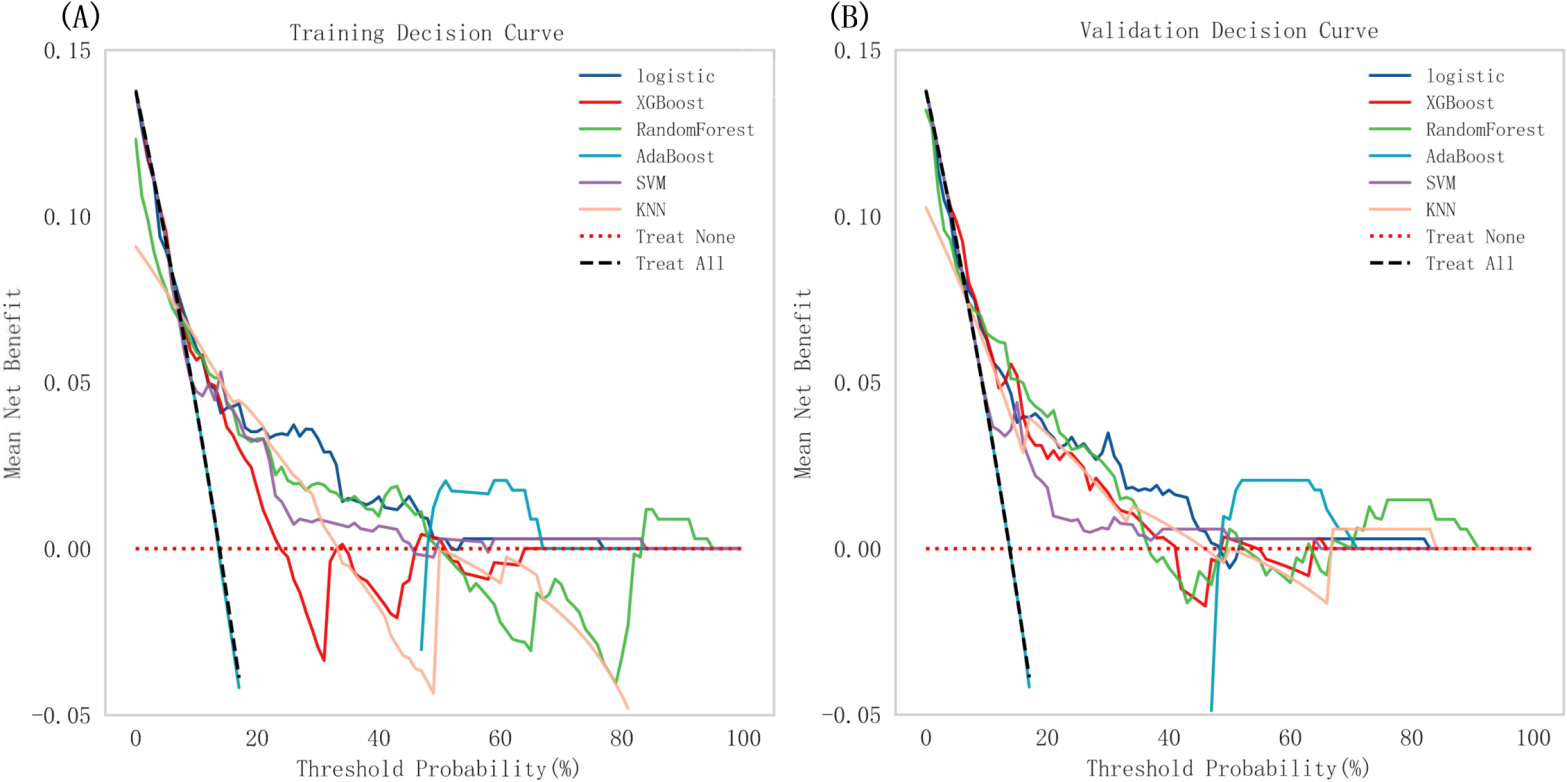
DCA curves of all models **(A)** Training cohort **(B)** Validation cohort.

Through the creation of receiver operating characteristic (ROC) curves (**Figure 3**) and the computation of various metrics (**Table 3**), we assessed the performance of all six machine learning models. The random forest (RF) model demonstrated the best overall performance with the highest accuracy (94.9%) and AUC (94.7%), followed by logistic regression (LR) (accuracy 91.2%, AUC 90.8%). The extreme gradient boosting (XGBoost) and adaptive boosting (AdaBoost) models showed moderate performance with accuracies of 89.5% and 87.3%, respectively. The support vector machine (SVM) achieved 86.4% accuracy, while the K-nearest neighbor (KNN) model had the lowest accuracy (79.8%) and AUC (78.5%). In terms of specificity, all models performed well (range 91.2%-96.7%), but the RF model achieved the highest sensitivity (83.5%) and F1 score (82.3%), indicating better balance between false positives and false negatives. Decision curve analysis (**Figure 4A**) confirmed that the RF and LR models provided the greatest net benefit across clinically relevant threshold probabilities. To assess these models’ clinical utility, DCA was used (**Figure 4A**). Across a wide range of threshold probabilities (approximately 10% to 80%), the random forest and logistic regression models achieved higher net benefits compared to the other models and to the “treat-all” and “treat-none” strategies. The random forest model demonstrated particularly favorable net benefit within the clinically relevant threshold range of 20% to 60%, where clinical decision-making is often most uncertain.

**Table 3:** Evaluation of every machine learning model.

Models	Accuracy (95%CI)	Sensitivity (95%CI)	Specificity (95%CI)	PPV (95%CI)	NPV (95%CI)	F1 score (95%CI)	Kappa (95%CI)
LR	0.825 (0.789-0.861)	0.575 (0.550-0.600)	0.865 (0.820-0.910)	0.42 (0.348-0.491)	0.927 (0.924-0.930)	0.48 (0.437-0.523)	0.38 (0.319-0.441)
XGBoost	0.793 (0.734-0.853)	0.727 (0.626-0.829)	0.803 (0.719-0.888)	0.402 (0.311-0.492)	0.95 (0.938-0.962)	0.502 (0.462-0.542)	0.392(0.327-0.457)
RF	0.949 (0.944-0.955)	0.835 (0.808-0.862)	0.968 (0.961-0.974)	0.806 (0.775-0.837)	0.974 (0.970-0.977)	0.82 (0.799-0.841)	0.79 (0.766-0.815)
AdaBoost	0.754 (0.692-0.816)	0.739 (0.682-0.795)	0.756 (0.676-0.836)	0.339 (0.289-0.388)	0.948 (0.942-0.954)	0.46 (0.418-0.501)	0.333 (0.270-0.395)
SVM	0.816 (0.785-0.847)	0.628 (0.590-0.666)	0.846 (0.812-0.880)	0.402 (0.343-0.461)	0.934 (0.926-0.942)	0.488 (0.438-0.538)	0.384 (0.318-0.450)
KNN	0.879 (0.860-0.898)	0.405 (0.326-0.484)	0.955 (0.920-0.989)	0.659 (0.476-0.843)	0.91 (0.901-0.919)	0.478 (0.455-0.502)	0.415 (0.388-0.443)

*XGBoost*, extreme gradient boosting; *LR*, logistic regression; *RF*, random forest; *AdaBoost*, Adaptive Boosting; SVM, support vector machines; KNN, K-nearest neighbor; *PPV*, positive predictive value; *NPV*, negative predictive value.

The RF model’s predictive ability was confirmed using the validation cohort, although it was less than that found with the training cohort. The ROC curves are displayed in **Figure 3B**. There is a good degree of agreement between the predictions and the measurements in the calibration curve (**Figure 4B**).

## 4 Discussion

Generally, most patients with PTC have a good prognosis. However, in some patients with PTC, cancer-related death and poor quality of life can occur once enlarged neck lymph nodes are detected (31). The recurrence of PTC leads to an increase in complications, including bleeding, hypothyroidism, damage to laryngeal nerves, and lymphatic leakage, compared to the initial surgery outcomes (32, 33). Therefore, predicting lymph node metastasis is important for surgeons to accurately guide neck dissection. Currently, if intraoperative pathology tests show no positive central lymph nodes, no additional LLND is performed, unless preoperative ultrasound-guided fine-needle aspiration cytology and imaging had revealed LLNM (15). However, LLNM preoperative tests show a high false-negative rate (34, 35) and their accuracy depends largely on the experience of the pathologists and ultrasound operators. Neglecting the extent of skip metastasis in PTC can result in insufficient lymph node dissection during surgery, which adversely affects patient outcomes. While relevant studies have proposed the clinical features of skip metastases and provided preliminary predictive frameworks, they share common limitations in their clinical significance and applicability.

In this study, we performed a comparative analysis of skip metastasis to fully characterize its clinical features. Machine learning is a type of classifier that learns to predict future data from past data. In our study, important factors in the various risk profiles were identified, further developed, and evaluated using six different machine learning models to evaluate their efficacy in detecting skip metastases, potentially offering greater clinical applicability.

The skip metastasis rate of LLNM in this cohort was 13.02% (47/361), compared to 1.6–25% in previous reports (24–26). The high incidence of skip metastases indicates their clinical relevance. A comparison of the lymph node metastasis types revealed notable disparities in maximum tumour size, presence of multiple tumors, and bilateral nature between the SKIP and CLNM groups, with significant differences between the SKIP and LLNM groups in terms of maximum tumour diameter and bilateral tumour size. Acknowledging these clinical features significantly aids in elucidating the reasons for skip metastasis and the pattern of lymph node metastasis in PTC.

The five features identified in our model have plausible biological explanations for their association with skip metastasis. Hashimoto’s thyroiditis may alter local lymphatic architecture through chronic lymphocytic infiltration, disrupting the typical stepwise metastasis from central to lateral compartments. Tumor multifocality and bilaterality increase total tumor burden and the probability of accessing alternative lymphatic drainage pathways, potentially bypassing the central compartment entirely. Regarding tumor size, the inverse relationship observed—smaller tumors associated with skip metastasis—suggests that skip metastasis may represent an early alternative pathway rather than a late-stage phenomenon. Finally, the number of central nodes removed reflects both surgical extent and underlying lymphatic anatomy; a low node yield may indicate incomplete sampling or anatomical variations that provide direct drainage to the lateral compartment.

This study identified five distinct indicators that predicted skip metastasis in PTC: the number of removed central neck lymph nodes, Hashimoto’s thyroiditis, presence of multiple tumour sites, presence of bilateral thyroid cancer, and tumour size. All these variables are readily determined in routine clinical practice. In this study the RF model was chosen to create a predictive model for skip metastasis to the lymph nodes in the lateral cervical area in patients with PTC. This model exhibited commendable efficacy, accuracy, effective patient differentiation, and robust clinical practicability. Additionally, its remarkable specificity and NPV indicate its ability to exclude patients without non-skip metastases. If the model predicted skip metastasis, its precision probability was estimated to be 94.9%. Consequently, when the model predicted unfavorable outcomes, individuals with metastases that did not include skip metastases could be effectively excluded. Additionally, the model exhibited a significant level of sensitivity (83.5%) and positive predictive accuracy (80.6%). Currently, a limited number of protocols support preventive LLND for cN0 thyroid cancer susceptible to skip metastasis, prompting the need for a model that focuses on identifying patients with non-skip metastasis and improving the specificity and NPV. Thus, when the model predicts a high probability of skip metastasis, either rigorous surveillance or intensified surgery may be considered, depending on the patient’s health status.

The potential causes for skip metastasis remain unclear. It has been noted that lymph metastasis can bypass lymph nodes in the absence of typical anatomical lymphatic channels (36, 37). Previous studies have indicated a link between the presence of Hashimoto’s thyroiditis and a less aggressive form of PTC^38^. Additionally, interventions such as extrathyroidal extension or neck treatment interfere with standard lymphatic routes and initiate skip metastases. Inadequate central and lateral lymph node sampling can miss nodes with metastases; minimal CLND may miss positive nodes and therefore the patient may appear to have skip metastasis.

This study has several limitations that should be acknowledged. First, the retrospective and single-center design inherently introduces potential selection bias and limits the generalizability of our findings. Although we included all eligible patients consecutively over a six-year period and applied strict data verification procedures, unmeasured confounders and institutional-specific surgical or pathological practices may still influence the results. For instance, the extent of central lymph node dissection and the pathological assessment of lymph nodes may vary across institutions, which could affect the reproducibility of our model. Second, while we used the Boruta algorithm to minimize overfitting and performed internal validation, the absence of external validation from an independent cohort remains a major limitation. Therefore, our model should be considered exploratory at this stage. Third, our model may have overlooked possible unaccounted confounders, such as molecular markers (e.g., BRAF^V600E^ mutation status) or detailed ultrasound features. Incorporating such variables may further improve predictive performance. To address these limitations, we plan to conduct a multicenter prospective study with standardized surgical and pathological protocols to externally validate our model and enhance its clinical applicability.

## 5 Conclusions

In conclusion, our study highlighted the importance of the number of removed central neck lymph nodes, Hashimoto’s thyroiditis, presence of multiple tumour sites, presence of bilateral thyroid cancer, and tumour size, all readily available clinical data, in identifying skip lymph node metastases in patients with PTC. We used machine learning to effectively predict the presence of skip metastases in patients with PTC. Through the development and evaluation of various machine learning models, it was determined that integrated learning methods, particularly RF models, performed well in internal validation with high accuracy.

## Data Availability

The original contributions presented in the study are included in the article/supplementary material. Further inquiries can be directed to the corresponding author.
